# Disparities in localized malignant lung cancer surgical treatment: A population‐based cancer registry analysis

**DOI:** 10.1002/cam4.5450

**Published:** 2022-11-17

**Authors:** Lohuwa Mamudu, Bonita Salmeron, Emmanuel A. Odame, Paul H. Atandoh, Joanne L. Reyes, Martin Whiteside, Joshua Yang, Hadii M. Mamudu, Faustine Williams

**Affiliations:** ^1^ Department of Public Health California State University, Fullerton Fullerton California USA; ^2^ Division of Intramural Research National Institute on Minority Health and Health Disparities, National Institutes of Health Rockville Maryland USA; ^3^ Department of Epidemiology Mailman School of Public Health, Columbia University New York New York USA; ^4^ Department of Environmental Health Sciences School of Public Health, University of Alabama at Birmingham Birmingham Alabama USA; ^5^ Department of Statistics Western Michigan University Kalamazoo Michigan USA; ^6^ Tennessee Department of Health Nashville Tennessee USA; ^7^ Department of Health Services Management and Policy College of Public Health, East Tennessee State University Johnson City Tennessee USA; ^8^ Center for Cardiovascular Risk Research, College of Public Health, East Tennessee State University Johnson City Tennessee USA

**Keywords:** lung cancer, surgical treatment, Tennessee, time to treatment initiation, treatment disparities

## Abstract

**Background:**

Lung cancer (LC) continues to be the leading cause of cancer deaths in the United States. Surgical treatment has proven to offer a favorable prognosis and a better 5‐year relative survival for patients with early or localized tumors. This novel study investigates the factors associated with the odds of receiving surgical treatment for localized malignant LC in Tennessee.

**Methods:**

Population‐based data of 9679 localized malignant LC patients from the Tennessee Cancer Registry (2005–2015) were utilized to examine the factors associated with receiving surgical treatment for localized malignant LC. Bivariate and multivariate logistic regression analyses, cross‐tabulation, and Chi‐Square (χ ^2^) tests were conducted to assess these factors.

**Results:**

Patients with localized malignant LC who initiated treatment after 2.7 weeks were 46% less likely to receive surgery (adjusted odds ratio [AOR] = 0.54; 95% confidence interval [CI] = 0.50–0.59; *p* < 0.0001). Females had a greater likelihood (AOR = 1.14; CI = 1.03–1.24) of receiving surgical treatment compared to men. Blacks had lower odds (AOR = 0.76; CI = 0.65–0.98) of receiving surgical treatment compared to Whites. All marital groups had higher odds of receiving surgical treatment compared to those who were single/never married. Patients living in Appalachian county had lower odds of receiving surgical treatment (AOR = 0.65; CI = 0.59–0.71) compared with those in the non‐Appalachian county. Patients with private (AOR = 2.09; CI = 1.55–2.820) or public (AOR = 1.42; CI = 1.06–1.91) insurance coverage were more likely to receive surgical treatment compared to self‐pay/uninsured patients. Overall, the likelihood of patients receiving surgical treatment for localized malignant LC decreases with age.

**Conclusion:**

Disparities exist in the receipt of surgical treatment among patients with localized malignant LC in Tennessee. Health policies should target reducing these disparities to improve the survival of these patients.

## Graphical abstract

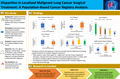

## 1 INTRODUCTION

Since 1987, lung cancer (LC) has been the leading cause of cancer deaths in men and women in the United States (U.S.).^1^, ^2^ Lung cancer is broadly classified into two types: (a) small cell lung cancer (SCLC), which accounts for approximately 15% of all cases, or (b) non‐small cell lung cancer (NSCLC), which accounts for approximately 85% of all cases. SCLC is rare compared to NSCLC; however, it is more aggressive and spreads faster with most cases diagnosed at an advanced stage.^3^ The American Cancer Society reported that people diagnosed with early‐stage, localized SCLC and NSCLC had 25% and 63% 5‐year survival rates, respectively, between 2010 and 2016.^1^ While there has been a decline in LC mortality rates, it still accounts for more deaths than breast, prostate, colorectal, and brain cancers combined.^1^, ^4^ Overall, LC is particularly lethal as cases tend to be diagnosed at a more advanced stage.^1^, ^5^

Currently, only 22.9% of LC cases nationally are diagnosed at an early stage in the U.S.^2^ LC incidence in Tennessee ranks among the five highest states in the country (3rd), at 74 cases per 100,000 people, compared to 58 per 100,000 nationally.^6^ The rate for Blacks (i.e., 68 per 100,000) in the state is significantly higher than the national average (60 per 100,000 population).^6^ Similarly, the rates for Whites (i.e., 77 per 100,000) is also higher than the national average for Whites (i.e., 63.1 per 100,000).^6^ Several risk factors, including sociodemographic factors, socioeconomic status (SES), genetics, and some lifestyle behaviors such as smoking can increase a person's chances to develop the disease.^7^, ^8^, ^9^, ^10^ However, having a risk factor does not necessarily mean one would get the disease.^11^ Cigarette smoking, the major risk factor for LC, accounts for approximately 80–90% of LC mortality.^12^ Moreover, LC rates have been higher in states that are historically known for growing tobacco, such as Tennessee.^13^ The current smoking rate in the state is higher than it is nationwide.^6^, ^14^ In 2020, almost 19.9% of adults 18 years and older in Tennessee were smokers, compared to 12.5% nationally, the 8th highest smoking rate among all states.^6^, ^14^

Surgical resection/treatment remains a well‐established treatment for localized malignant LC and found to improve survival rates.^2^, ^15^, ^16^ Overall, surgical treatment significantly improves survival compared to chemotherapy and radiotherapy.^4^ Nevertheless, disparities in healthcare and LC outcomes still exist.^17^ Prolonged delays in initiating treatments such as surgical resection, radiotherapy, or chemotherapy, have been shown to expedite the progression of the disease.^18^ Delaying the initiation of treatment can cause the disease to metastasize, leading to poor prognosis and survival. There is no definitive answer to how long or fast LC takes to metastasize because it depends on several factors, including the type, location, and stage of the cancer. Although it is unknown how long a person should wait after diagnosis to start treatment, it remains clear that early treatment initiation after diagnosis improves survival outcomes.^19^, ^20^, ^21^ On average, the duration of delay in treatment initiation of LC varies from 14 to 56 days among studies.^22^, ^23^ Previous studies on delayed surgery among LC patients have suggested different social determinants for the variability in delay.^24^, ^25^, ^26^ Race/ethnicity provides a major explanation for the disparities, with Blacks more likely to experience late treatment for LC and other types of cancers compared to Whites.^17^, ^25^, ^27^, ^28^, ^29^, ^30^ Socioeconomic status (SES) also plays a critical role. Previous studies have found that sociodemographic variables such as income, education, and type of insurance influenced an individual's ability to receive healthcare treatment for cancer.^26^, ^31^, ^32^, ^33^ High SES is associated with more favorable outcomes and vice‐versa.^16^, ^17^, ^34^ As such, the purpose of this study is to investigate the association of sociodemographic characteristics, type of insurance coverage, and time to initiation of treatment of localized malignant LC with the likelihood of receiving surgical treatment among patients in Tennessee.

## 2 MATERIALS AND METHODS

### 2.1 Data source and study population

We obtained Tennessee Cancer Registry (TCR) data from all Tennessee residents diagnosed with histologically confirmed LC as the primary site (C340‐349) of diagnosis and histological type 8000–9053 codes by the International Classification of Diseases for Oncology, Third Edition (ICD‐O‐3) from January 1, 2005 to December 31, 2015. The TCR is a population‐based, central cancer registry serving the citizens of Tennessee and was established by Tennessee law to collect and monitor cancer incidence (https://www.tn.gov/health/health‐program‐areas/tcr/tennessee‐cancer‐registry‐data.html). A total of 43,395 lung cancer cases were diagnosed between 2005 and 2015, of which 9679 were at the localized stage. In this study, we included individuals who were diagnosed with localized malignant stage LC and received treatment/procedure within 12 months. We focused on localized stage disease because surgical procedure is often the recommended treatment for patients whose disease has not metastasized or spread to distant organs or lymph nodes.^35^ Data used for this analysis are restricted but available by request to the Tennessee Department of Health‐TCR (https://www.tn.gov/education/data/data‐downloads/request‐data.html). All analytical files are available by reasonable request per Tennessee Department of Health approval. The Tennessee Department of Health Institutional Review Board approved the study protocol.

### 2.2 Dependent variable of interest

The dependent or outcome variable for this study is a dichotomized variable of whether the localized malignant LC patient received surgical treatment or procedure. It was coded 1 for patients who had surgery and 0 for those who did not.

### 2.3 Independent variables/covariates

Individual‐level sociodemographic variables obtained from TCR included race, age at diagnosis, marital status, and county of residence. Other factors included type of health insurance, date of LC diagnosis, and date treatment commenced. Following the criteria from the National Institute of Aging, age was divided into five groups as: <45, 45–54, 55–64, 65–74, and ≥ 75 years,^36^ and race as Black or White. Marital status was categorized as single/never married, married/common‐law, divorced/separated, and widowed. Type of insurance was classified as public (Medicaid, Medicare, Indian Health Service, Veterans Affairs) to due restriction from TCR; private (fee for service, health maintenance organization [HMO], managed care, or preferred provider organization [PPO]); and self‐pay or uninsured. Tennessee is a unique state with 95 counties. Fifty‐two out of 95 counties are in the Appalachian region and the remaining 43 are in the non‐Appalachian region. Most counties in the Appalachian region are largely considered economically disadvantaged.^37^ As a result, place/county of residence was characterized into two groups based on whether the patient resided in an Appalachian or non‐Appalachian county in Tennessee. Assessment of duration of wait time/or initiation to treatment was dichotomized using the median number of weeks delay between diagnosis and the first treatment as ≤2.7 weeks and >2.7 weeks. Two point seven weeks (2.7 weeks) represents our sample median, which is robust and resistant to outliers, and also falls within the time considered as treatment delay for LC among studies.^22^, ^23^ Time to initiation of treatment was used as the primary independent variable and the remaining variables as confounding covariates.

### 2.4 Statistical analysis

Descriptive statistics were generated using frequencies and percentages as well as the median and standard deviation for age at diagnosis and time to treatment initiation. We then performed bivariate and multivariate analysis using an adjusted logistic regression model to examine the association between receiving localized LC surgical treatment and sociodemographic characteristics, type of health insurance coverage, and wait time between diagnosis and treatment initiation. Chi‐square (χ ^2^) test, Likelihood Ratio test, Fisher's Exact test, and cross‐tabulation were used to further assess the statistical difference and relationship between receiving surgical treatment and the time to initiation of treatment after diagnosis. Results are reported using the adjusted odds ratios (AORs) along with 95% confidence intervals (CIs) and a statistical significance level of *p*‐value <0.05. All analyses were conducted using IBM SPSS Statistics 28 Premium and SAS statistical software, version 9.4 (Cary, NC).

## 3 RESULTS

### 3.1 Localized lung cancer patient sample characteristics

A total of 9679 localized stage malignant LC cases were diagnosed in Tennessee between January 2005 and December 2015. Of these, 62.56% (6055) patients received surgical treatment compared to 3624 (37.4%) who did not. The median age at diagnosis was 67 years. The sample was approximately evenly distributed between males (50.4%; *n* = 4879) and females (49.6%; *n* = 4800). The majority of the patients were White (91.3%; *n* = 8832), married/common law (57.6%; *n* = 5575), resided in an Appalachian county (55.1%; *n* = 5409), and started treatment within 2.7 weeks (58.1%; *n* = 5644) after diagnosis. Most of the patients had public health insurance, (74.3%; *n* = 7193), followed by private health insurance coverage (23.5%; *n* = 2271) whereas less than 3% (*n* = 936) reported no health insurance coverage. Patients' sociodemographic characteristics are displayed in Table 1.

**TABLE 1:** Localized malignant lung cancer patient sample characteristics and bivariate statistical significance with surgical treatment (*N* = 9679)

Characteristics	Total (N)	Percent (%)	Median	SD
Age at diagnosis	9679	—	67	10.52
Time to Treatment Initiation (Day)	—	—	19	33.06
Time to Treatment Initiation (Week)	—	—	2.71	4.72
				***p*‐value**
**Sex**				**0.001**
Male	4879	50.4		
Female	4800	49.6		
**Age at diagnosis**				**<0.001**
<45	134	1.4		
45–54	795	8.2		
55–64	2225	23.0		
65–74	3804	39.3		
75+	2721	28.1		
**Race**				**<0.001**
White	8832	91.2		
Black	847	8.8		
**Marital status**				**<0.001**
Single/Never Married	936	9.8		
Married/Common Law	5575	57.6		
Divorced/Separated	1271	13.1		
Widowed	1897	19.6		
**County of residence**				**<0.001**
Non‐Appalachian	4270	44.1		
Appalachian	5409	55.1		
**Type of health insurance coverage**				**<0.001**
Self‐Pay/Uninsured	215	2.2		
Public	7193	74.3		
Private	2271	23.5		
**Surgical treatment**				‐
Yes	6055	62.6		
No	3624	37.4		
**Time to treatment initiation**				
≤2.7 weeks	5644	58.1		**<0.001**
>2.7 weeks	4069	41.9		

*Note*: Statistical analysis performed = Descriptive and bivariate analysis. Bold = Statistically significance (*p* < 0.05). Private Insurance (Fee for services, Health Maintenance Organization [HMO], Managed care, Preferred provider organization [PPO]). Public Insurance (Indian Health Service, Medicaid, Medicare, Veterans' Affairs).

Abbreviation: SD, Standard deviation.

The bivariate analysis showed a statistically significant relationship between receiving surgical treatment for localized malignant LC and sex, age, race, county of residence, health insurance coverage, and time to treatment initiation, all given by *p*‐value <0.05 level of significance.

### 3.2 Assessment of likelihood of localized malignant lung cancer surgical treatment

All the variables were significantly associated with the receipt of LC surgery, including time to treatment initiation (See Table 2). Overall, individuals with localized LC who started treatment after 2.7 weeks were 46% less likely to receive surgical procedure (AOR = 0.54; 95% CI: 0.50–0.59; *p* = <0.0001) after controlling for other factors, and those living in the Appalachian county of Tennessee were 35% less likely to undergo surgery for localized malignant LC (AOR = 0.65; 95% CI = 0.59–0.71; *p* = <0.0001). Similarly, Tennessee females were more likely than males to receive surgery (AOR = 1.14; 95% CI: 1.03–1.24; *p* = 0.007). Compared to self‐pay/uninsured patients, individuals with private insurance coverage (i.e., fee for service) and public insurance (i.e., Medicaid, Medicare, etc.,) had 109% (AOR = 2.09; 95% CI: 1.55–2.82; *p* = <0.0001) and 42% (AOR = 1.42; 95% CI: 1.06–1.91; *p* = <0.019) increased odds, respectively to receive surgical procedure whereas Blacks patients in Tennessee had 24% less likelihood to receive surgical treatment (AOR = 0.76; 95% CI = 0.65–0.90; *p* = 0.001) relative to White patients. Marital status was also significantly associated with the receipt of LC surgical treatment. Divorced/separated patients (AOR = 1.78; 95% CI: 1.53–2.03; *p* = <0.0001) were more likely than widowed (AOR = 1.30; 95% CI: 1.09–1.56; *p* = 0.004), and married/common law (AOR = 1.22; 95% CI: 1.02–1.45; *p* = 0.023) to have surgical treatment. The age at diagnosis was also significantly associated with LC surgical procedure. However, the likelihood of receiving surgical treatment decreased with increasing age. For instance, patients <45 years had 378% increased odds (AOR = 4.78; 95% CI: 2.94–7.76; *p* = <0.0001) of having surgery compared to individuals aged 65–74 years who had 108% increased odds (AOR = 2.08; 95% CI: 1.88–2.31; *p* = <0.0001). See Table 2 for details.

**TABLE 2:** Likelihood of localized malignant lung cancer surgical treatment (*N* = 9679)

		95% confidence interval		
Characteristics description	AOR	Upper	Lower	*p*‐value
**Sex**				
Male [Ref.]	—	—	—	—
Female	1.14	1.03	1.24	**0.007**
**Age at diagnosis**				
<45	4.78	2.94	7.76	**<0.0001**
45–54	3.19	2.60	3.92	**<0.0001**
55–64	2.37	2.06	2.72	**<0.0001**
65–74	2.08	1.88	2.31	**<0.0001**
≥75 [Ref.]	—	—	—	—
**Race**				
White [Ref.]	—	—	—	—
Black	0.76	0.65	0.98	**0.001**
**Marital status**				
Single/Never Married [Ref.]	—	—	—	—
Married/Common Law	1.22	1.02	1.45	**0.023**
Divorce/Separated	1.78	1.53	2.03	**<0.0001**
Widowed	1.30	1.09	1.56	**0.004**
**County of residence**				
Non‐Appalachian [Ref.]	—	—	—	—
Appalachian	0.65	0.59	0.71	**<0.0001**
**Type of health insurance coverage**				
Self‐Pay/Uninsured [Ref.]	—	—	—	—
Private	2.09	1.55	2.82	**<0.0001**
Public	1.42	1.06	1.91	**0.019**
**Time to treatment initiation**				
≤2.7 weeks [Ref.]	—	—	—	—
>2.7 weeks	0.54	0.50	0.59	**<0.0001**

*Note*: Statistical analysis performed, Multivariate logistic regression analysis. Bold = Statistically significance (*p* < 0.05).

Abbreviations: AOR, Adjusted odds ratio; Ref, Reference.

### 3.3 Assessment of statistical significance between localized malignant lung cancer surgical treatment and time to surgical treatment initiation

**FIGURE 1:**
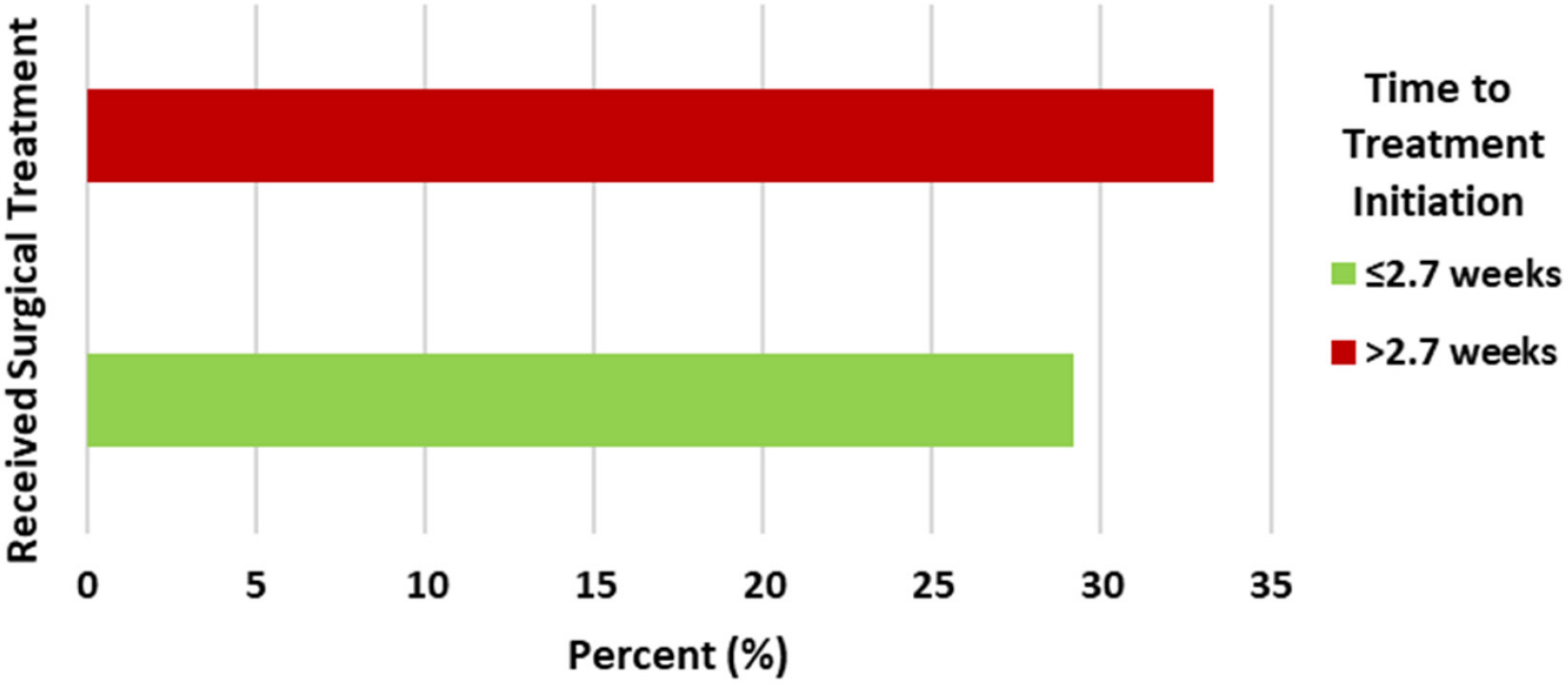
Localized malignant lung cancer surgery by time to treatment initiation (cutoff median time of 2.7 weeks).

Of the 6055 (62.6%) localized LC patients who received surgical treatment, 2831 (46.8%) spent at most 2.7 weeks before receiving surgical treatment and 3224 (53.2%) waited more than 2.7 weeks before receiving surgical treatment (See Table 3 and Figure 1 for more detail). After 2.7 weeks of localized LC diagnosis, there was a proportionately higher number of patients (68.2%) who received other treatment compared with 53.2% of those who received surgery. On the contrary, there was proportionately higher number of patients (46.8%) who received surgical treatment before 2.7 weeks compared with 31.8% of those who received other treatment. To validate the association of time to treatment initiation with choice of surgical treatment, we conducted a χ ^2^ independent test between the independent variable only (time to treatment initiation) and the outcome variable, which showed further evidence of statistical significance association (χ ^2^ = 208.12, *p* < 0.001) between time waited to initiate treatment after diagnosis and whether an individual would undergo surgical treatment (See Table 3).

**TABLE 3:** Statistical difference and relationship of localized malignant lung cancer surgical treatment and time to initiation of surgical treatment

		Received surgical treatment			Two‐sided χ^2^, likelihood ratio, and Fisher's exact test	
		No *n* (%)	Yes *n* (%)	Total *N* (%)	Statistic values	*p*‐value
**Time to treatment initiation**	**≤2.7 weeks**	1154 (11.9)	2831 (29.2)	3985 (41.2)		**<0.001**
		31.8%	48.6%		208.1	
	**>2.7 weeks**	2470 (25.5)	3224 (33.3)	5694 (58.8)	211.2	
		68.2%	53.2%			
	T**otal**	3624 (37.4)	6055 (62.6)	9679		

*Note*: Statistical test performed = Pearson χ^2^, Likelihood ratio, and Fisher's exact test of association. Bold = Statistically significance (*p* < 0.05).

## 4 DISCUSSION

Surgical resection remains the most recommended form of treatment for lung cancer, especially, non‐small cell lung cancer.^38^, ^39^ Previous studies have shown that surgical treatment can result in a 70–90% 5‐year survival rate for NSCLC.^19^, ^20^, ^21^ However, racial and geographical disparities exist in the receipt of LC surgical treatment in Tennessee. Additionally, Tennessee covers a substantial portion of the Appalachian region, designated by the National Cancer Institute as a priority area due to being medically underserved, higher rates of cancer, and significant socioeconomic disadvantages.^26^, ^31^, ^32^, ^33^, ^40^ It is imperative to examine factors associated with surgical treatment of patients diagnosed with localized malignant LC in areas of Tennessee to address the underlying disparities.

This study found significant association between receipt of surgical treatment and sociodemographic factors (sex, race, age at diagnosis, marital status, and county of residence), health insurance type, and delayed treatment after 2.7 weeks. This finding is consistent with previous studies.^41^, ^42^, ^43^, ^44^, ^45^ Additionally, it was found that Black patients, individuals residing in Appalachian Tennessee, and those who initiated treatment later (i.e., >2.7 weeks) had significantly decreased odds of receiving surgical treatment. Consistently, Soneji et al.^44^ reported that Black patients diagnosed with early stage LC were less likely to receive surgical treatment compared with their White counterparts. The authors further noted that Black patients are more likely not to be recommended for surgery.^44^ Likewise, in a study that assessed healthcare providers' biases and racial disparities in LC patients recommended surgical therapy, the authors attributed the lower odds of receiving treatment between Black and White (OR = 0.43, 99% CI = 0.36–0.52) to healthcare system biases, distrust, beliefs, and perceptions about the disease.^46^ Although we did not assess physician recommendations of surgery for Black patients, we found Blacks were 24% less likely to undergo surgical treatment. Another study using the linked National Longitudinal Mortality Study and Surveillance, Epidemiology, and End Results^17^ data found the hazard ratio for cancer‐specific mortality was significantly higher among Blacks than Whites after adjusting for age, sex, and tumor stage, and further after controlling for socioeconomic factors, and treatment. Clearly, Blacks are at higher risk of not receiving recommended surgical treatment for LC, even though surgical treatment can prolong the length of their survival. Developing LC treatment policies that target Black communities can minimize such racial disparities in the treatment of LC.

Advanced age is strongly a risk factor for poor treatment and outcomes for cancer patients, especially surgery.^47^, ^48^, ^49^ In support of this evidence, it was found that the likelihood of patients with localized malignant LC receiving surgical treatment decreases with increasing age. Similarly, Samson et al.^50^ used the National Cancer Data Base to investigate the effects of delayed surgical treatment on short and long‐term outcomes in clinical stage I NSCLC. They found delay in surgery to be associated with increasing age, not being White, treatment at an academic center, and living in urban location.^50^ Additionally, a previous study that investigated NSCLC using data from the Kentucky Cancer Registry from 2004 to 2014 found that non‐married patients were less likely to receive surgery,^51^ which is supported by our finding of an increased likelihood of married patients with localized malignant LC receiving surgical treatment compared to single/never married individuals. Also, a feasibility LC mobile bus that screened at‐risk rural patients over 12 months in Tennessee noted that rural hospital closures and healthcare provider shortages present many challenges for patients.^52^ Consistent with these, a retrospective cohort study by Johnson et al. found that NSCLC patients residing in rural Georgia had lower odds of receiving surgery.^53^ It was found in our study that patients residing in Appalachian Tennessee, largely regarded as the rural region of Tennessee, are 35% less likely to receive surgical treatment for localized malignant LC compared with those residing in the non‐Appalachian region. Inadequate socio‐economic factors such as insufficient healthcare access may play a key role in causing this problem. Improving them can be a mitigating effect. However, further studies may be needed to investigate and better understand the root cause of the problem.

Regarding the type of health insurance coverage, the findings in this study show individuals with insurance, private or public, had increased odds of receiving surgical procedures compared to self‐pay/uninsured, although those with private health insurance are more likely than individuals with public health insurance coverage to receive surgical treatment. Consistent with our findings, Maguire et al.^54^ used the California Registry data from 2012–2014 to examine the influence of health insurance type on receipt of systematic treatment of NSCLC and found patients with Medicaid or other public insurance were significantly less likely to receive any systemic treatments compared with those privately insured. Other studies consistent with our findings also reported that patients diagnosed with NSCLC who are uninsured or covered by Medicaid are less likely to receive recommended surgical treatments than patients with private insurance.^55^, ^56^, ^57^ Some of the suggested reasons why uninsured or publicly insured patients may have a decreased likelihood of receiving systemic or recommended treatments for LC include inadequate Medicaid providers leading to long waiting times to see one, low reimbursement levels leading to late or long awaiting care, and high prices of cancer drugs, in particular, novel medications with formulary restrictions.^56^, ^58^ Increasing the number of public health insurance providers such as Medicaid and enacting policies to reduce the high prices of LC drugs can help reduce the waiting time to receive recommended surgical treatment to improve the survival of patients.

This study found that localized LC patients who wait more than 2.7 weeks after diagnosis had a 46% decreased likelihood of receiving surgical treatment. Meanwhile, despite the significant improvement in LC screening detection, therapeutic, and oncological management of advanced stage LC, surgical treatment of early‐stage NSCLC can result in at least a 70% 5‐year survival rate.^59^ There are several reasons why LC surgical treatment delay beyond 2.7 weeks might happen. Salomaa et al. found in a retrospective study of 132 patients that delay from diagnosis to treatment of LC occurred as a result of delay by patients, the first appointment of General Practitioner (GP) delay, GP writing for referral delay, from referral to the first visit of specialist delay, and finally, treatment.^60^ These delays or procedures patients go through can result in aggravating the LC, and as a result, surgery may no longer be the ideal form of treatment. A multidisciplinary team approach and rapid access to carefully planned investigations can help shorten the delay times by the health care institutions. Tackling patients' delayed action may be more difficult. However, patient education on the risk of delaying treatment can create awareness and help reduce patients' delay time.

The objective of this study is part of a broader effort to understand the dynamics of cancer‐related health disparities in Tennessee, strengthen a culture of collaboration to reduce cancer‐related health disparities, and address public policy as it relates to Tennessee's cancer‐related health disparities.^61^ The findings show disparities still exist among patients diagnosed with localized malignant LC in receiving surgical treatments. Black patients, those living in Appalachian Tennessee, and those who started treatment after 2.7 weeks have a lower odds of receiving surgical resection. Developing health policies to eliminate these disparities can improve the survival rate and reduce the mortality of patients diagnosed with localized malignant LC.

Our study findings carry several policy implications for local public health officials and policymakers. Early diagnosis, treatment initiation, and recommended surgical treatment are essential for better survival outcomes. Therefore, early testing and early treatment initiation go hand in hand. As noted in the Tennessee Cancer Plan, the goal is to detect the disease early and initiate timely treatment, and our findings support such an effort. Our findings regarding the health insurance status of patients may warrant further research. Future studies should consider a greater variety of sample data to determine their association with other treatment types like radiotherapy, chemotherapy, etc., and how that influence the choice of surgery and impact on mortality. Taking our findings further to incorporate mortality data will help create a better understanding of the state of LC treatment outcomes in Tennessee and will help the state monitor progress in achieving the goals laid out in the Tennessee Cancer Plan.

## 5 LIMITATIONS

There are some limitations of this study. First, the cross‐sectional data limit our ability to generate any causal inference between surgical treatment and the included factors. Secondly, we were constrained by the retrospective administrative variables available to us. For instance, cancer registries do not collect SES data (e.g., income, education) and the quality of treatment received by patients. Additionally, some demographic variables collected may not be up to date since these variables are only collected at the time of diagnosis. Further, the results of the study may not be generalizable to other states or the entire U.S. population. Nevertheless, the findings are essential because they provide a better understanding of patients diagnosed with localized malignant LC and the factors associated with the timely initiation of surgical treatment in Tennessee.

## 6 CONCLUSION

Racial and geographical disparities exist in the receipt of LC surgical treatment in Tennessee. Men, Blacks, elderly patients, people residing in Appalachian Tennessee, uninsured individuals, and those who initiated treatment later were less likely to receive surgical treatment. Other reasons which may contribute to underutilization of surgical treatment services for localized malignant LC in Tennessee warrant further study. Furthermore, public health interventions should focus on reducing these disparities to improve the survival of lung cancer patients in Tennessee.

## AUTHOR CONTRIBUTIONS

**Lohuwa Mamudu:** Conceptualization (lead); data curation (lead); formal analysis (lead); investigation (equal); methodology (lead); software (lead); visualization (lead); writing – original draft (lead). **Bonita Salmeron:** Writing – original draft (equal). **Emmanuel A Odame:** Writing – original draft (equal). **Paul H Atandoh:** Validation (lead); visualization (equal); writing – review and editing (equal). **Joanne L Reyes:** Writing – original draft (equal). **Martin Whiteside:** Investigation (equal); writing – review and editing (equal). **Joshua Yang:** Writing – review and editing (equal). **Hadii Mamudu:** Writing – review and editing (equal). **Faustine Williams:** Conceptualization (equal); investigation (lead); methodology (equal); project administration (lead); resources (lead); supervision (lead); validation (equal); visualization (supporting); writing – review and editing (lead).

## CONFLICT OF INTEREST

The authors declare no conflict of interest.

## ETHICS APPROVAL

The research protocol and access to the data for this study went to full review by the Tennessee Department of Health Institutional Review Board (IRB) and was approved on 01 February 2018 (TDH‐IRB‐1057486). The National Institutes of Health—Intramural Research Program IRB—Human Research Protections Program—Office of Human Subjects Research Protections determined that our protocol did not involve human subjects and was excluded from IRB review (18‐NIMHD‐00722).

## Data Availability

Data used for this analysis are restricted but available by request to the TDH, Tennessee Cancer Registry, https://www.tn.gov/education/data/data‐downloads/request‐data.html.
